# Parental correlates of adolescent gambling behavior: a study on 12–14 years old students in Italy

**DOI:** 10.3389/fpsyg.2025.1563936

**Published:** 2025-09-09

**Authors:** Erica Viola, Emina Mehanović, Marco Martorana, Mariaelisa Renna, Alberto Sciutto, Giulia Giraudi, Maria Ginechesi, Claudia Vullo, Serena Vadrucci, Chiara Andrà, Adalgisa Ceccano, Pietro Casella, Fabrizio Faggiano, Federica Vigna-Taglianti

**Affiliations:** ^1^Department of Sustainable Development and Ecological Transition, University of Piemonte Orientale, Vercelli, Italy; ^2^Department of Psychology, eCampus University, Novedrate, Italy; ^3^Department of Translational Medicine, University of Piemonte Orientale, Novara, Italy; ^4^Department of Statistics, Computer Science, and Applications “Giuseppe Parenti” (DiSIA), University of Florence, Florence, Italy; ^5^Department of Mental Health, Addiction Unit, ASL Roma 1, Rome, Italy; ^6^Department of Prevention, Hygiene and Public Health Unit, ASL Città di Torino, Turin, Italy; ^7^Epidemiology Unit, ASL Vercelli, Vercelli, Italy

**Keywords:** gambling, adolescents, parents, correlates, mediation, Italy

## Abstract

**Objective:**

This study aims to elucidate the role of parental factors on adolescents' gambling in a sample of Italian students, and to identify the mediating factors of the relationship between parental gambling and adolescents' gambling.

**Methods:**

This is a secondary study based on data collected in the baseline survey of the experimental controlled trial “GAPUnplugged”. The analytical sample included 1,848 students 12–14 years old who participated in the study in Piedmont and Lazio Regions in Italy. Multilevel mixed-effect regression models were used to estimate the associations between correlates and the probability of adolescents' gambling. Mediation analysis was conducted to test the mediating effect of personal factors on the relationship between parental and adolescents' gambling.

**Results:**

Overall, 55.7% of students reported gambling in the last 12 months. In the multivariate regression model, gambling with parents and parental permissiveness to gamble were the strongest correlates of adolescents' gambling. Parental gambling, parental permissiveness to use licit substances and perception of friends' gambling were also significantly associated with adolescents' gambling. Performance beliefs, attitudes toward gambling, and sensation-seeking emerged as potential mediators of the relationship between parental gambling and adolescents' gambling.

**Conclusions:**

Parental factors appear to be important correlates of gambling behavior among adolescents. These results provide insights into the complex dynamics influencing adolescent gambling behavior and emphasize the importance of targeted interventions and parental guidance to promote healthier decision-making and mitigate adolescent gambling problems.

## 1 Introduction

Adolescent gambling is a serious public health problem (Armitage, 2021; Delfabbro et al., 2016). The first involvement in gambling activities appears to occur in early adolescence, between 11 and 12 years of age (Gambling Commission, 2023; Derevensky et al., 2019; Westphal et al., 2000), however scarce data are available on gambling prevalence at such an early age. Over the past 12 months, 22% of European 16-year-old students engaged in gambling, and among them, 5% exhibited problematic gambling behavior (ESPAD Group, 2020). In Italy, 32% of students gambled at least once in the last 12 months, and 3.9% of them had problem gambling (ESPAD Group, 2020). The prevalence of gambling behavior is decreasing over the years (HBSC, 2022). However, it remains a serious issue deserving deeper identification of risk factors and implementation of appropriate interventions.

Family plays a crucial role in the development of habits and behaviors among adolescents: parental behavior provides a strong role model for children behavior (Bandura, 1977, 1986). Indeed, the association between parental gambling and the risk of adolescents' gambling has been well-documented (Delfabbro et al., 2005; Gupta and Derevensky, 1997; Hardoon et al., 2004; Langhinrichsen-Rohling et al., 2004; McComb and Sabiston, 2010; Vachon et al., 2004). Moreover, some parental practices such as low parental monitoring and support may act as risk factors for adolescents' pro-gambling attitudes and engagement in gambling-related activities (Hardoon et al., 2004; Lee et al., 2014; McComb and Sabiston, 2010; Molinaro et al., 2014; Vachon et al., 2004).

The role of family socioeconomic status in influencing gambling behavior among adolescents is less clear. In most previous studies, family high socioeconomic status acted as risk factor, with dose-response association between the possession of money (e.g., higher weekly income) and the probability of adolescent gambling (Moñino-García et al., 2022; Buja et al., 2019; Welte et al., 2008). However, other studies found that adolescents who gamble are more likely to come from lower social classes (Griffiths and Wood, 2000; Barnes et al., 2005).

Finally, personal risk factors for adolescents' gambling have been extensively studied in the literature, including attitudes, beliefs and expectancies, risk perceptions, self-esteem, depression, impulsiveness, sensation-seeking and many others (Dussault et al., 2011; Hurt et al., 2008; Dowling et al., 2017; Derevensky and Gilbeau, 2015; Kaltenegger et al., 2019; Reardon et al., 2019; Renna et al., 2025). To our knowledge, no prior studies investigated the mediation effect of the relationship between parental factors and adolescents' gambling.

A deep understanding of parental factors related to gambling behaviors in early adolescents is needed to correctly address the problem and develop prevention interventions. This study aims to elucidate the role of parental factors, and particularly socio-economic status, parental behaviors, and permissiveness, on adolescents' gambling in a sample of 12–14 years old Italian students. A second aim is to test the mediating effect of adolescent beliefs, attitudes, risk perceptions, impulsiveness and sensation-seeking on the relationship between parental gambling and gambling with parents, and adolescents' gambling.

## 2 Methods

### 2.1 Study design and sample

This is a secondary study based on data collected in the baseline survey of the experimental controlled trial “GAPUnplugged” (Vigna-Taglianti et al., 2024). The baseline survey involved 1874 students 12-14 years old of 29 secondary schools located in the territories of nine National Health Service (NHS) districts (Rome, Alessandria, Torino 3, Torino 5, Vercelli, Cuneo 1, Cuneo 2, city of Torino, Novara) of Piedmont and Lazio Regions in Italy between November 2022 and January 2023. The analytical sample of the present study included 1,848 students who provided the answer to the question of gambling in the last 12 months.

### 2.2 Data collection

A self-completed anonymous questionnaire was used to collect information on sociodemographic characteristics, substance use, gambling behaviors, beliefs, attitudes and risk perceptions toward gambling, the perception of peers' and friends' substance use and gambling, friend's approval of gambling, parental gambling, parenting monitoring, parental support, disappointment of parents, parental permissiveness toward use of licit substances and gambling, refusal skills, school climate, relation with mathematics and grades, impulsiveness, self-esteem, antisocial behaviors (e.g., violence, stealing) and sensation-seeking. Only students whose parents or caregivers gave consent to participate were involved in the study. Before the administration of questionnaires, information on the study was provided to the pupils and consent to participate was asked. The questionnaire was developed *ad hoc* and included previously validated questions derived from the Unplugged evaluation survey (https://eudap.eu/), EDDRA data bank of EMCDDA (https://www.euda.europa.eu/) and other international sources and projects (ESPAD, HBSC, Project ALERT, RATING Swedish cohort, SOGS-RA Italian validated version, BSSS Italian version). To preserve confidentiality of the data a 9-digit individual code was self-generated by the student. The questionnaires were filled in by students in the classroom during the school time through online application. In cases of lack of computers or problems of connection, the researchers administered to the students the paper version of the questionnaire.

### 2.3 Measures

Individual socio-demographic information included gender, age (based on birth date), languages spoken in family, family composition (living with “Both parents”, “One parent”, and “Others”), and indicators of socioeconomic status (father and mother occupation, and family holidays). Mother and father occupation was assessed by asking “What job does your mother do?” and “What job does your father do?”. Based on the answers we created two new indicators of socioeconomic status. The first one was “Employment status” categorized as “Work”, “Doesn't work” and “Retired” for each parent separately. The second one was “Parental income” categorized as “Two salaries”, “One salary” and “No salary” depending on each parents' employment status. Family holidays were measured by using the question “During the last year, how many times did you travel away on holidays with your family?” with possible answers “Never”, “Once”, “Twice” and “More than twice”.

A 3-item question assessed how often adolescents received money from their parents, allowing the answers “Almost never”, “Rarely”, “Sometimes” and “Often”.

Gambling behavior was investigated by asking students if they gambled (scratch cards, lottery, bingo, slot machines, sport betting, event betting, poker, cards) during the last 12 months, with response categories ranging on a scale from 0 to 13 times or more for each specific game. A unique variable of gambling behavior was created, and all the answers were summed up into a dichotomous indicator “Yes” and “No”.

Exposure to parental gambling was measured using the question “Does any of your parents gamble?” allowing the following answers “Yes, only father”, “Yes, only mother”, “Yes, both”, “No” and “Don't know”. The answers were then recategorized into “Yes”, “No” and “Don't know”. Gambling with family components was measured by asking “Have you ever gambled together with your father, mother, siblings, other relatives?” with possible answers “Never gambled in general”, “Never gambled with father, mother, siblings, and other relatives”, “Sometimes” and “Often” for each family member separately.

Perceived parental permissiveness toward licit substances was assessed by asking students if their parents would allow them smoking and drinking alcohol (separate questions), with possible responses “Would not allow at all”, “Would not allow at home”, “Would allow”. Perceived parental permissiveness toward gambling allowed responses “Would not allow” and “Would allow”. Parental monitoring was investigated by asking students to provide responses on the following statements: “My parents set clear rules” and “My parents know where I am in the evening”. Parental disappointment was explored through the statement “It is very important for me not to disappoint my parents”. Parental support was investigated through the statement “I can easily get support from my father and/or mother”.

Questions on the perceived number of friends gambling in presence and online allowed the answers “None”, “Less than half of them”, “About half of them”, “More than half of them” and “All of them”. Answers were collapsed into a dichotomous variable of friend's gambling “Yes” and “No”.

Performance beliefs toward gambling were assessed through the following statements “I have an ability to predict my gambling winnings”, “Gambling is a sure way of becoming rich”, “If I gamble often, I have higher probability of winning”, “Winning and losing in gambling depends only on chance”, “Those who play sport have higher probability of winning the sports betting”, “If I come close to winning now, next time I will win” and “In the lottery, if a number doesn't come out for a long time, it will certainly come out soon”. The reliability of the scale was good (Cronbach's alpha α = 0.78). Positive attitudes toward gambling were assessed through the items “I find it funny”, “I find it enthusiastic” and “I will become rich”. The reliability of the scale was good (Cronbach's alpha α = 0.68). Negative attitudes were assessed through the items “I find it risky”, “I think it could become a habit”, “I could lose the money”. The reliability of the Cronbach's alpha was α = 0.41. Risk perceptions were measured by using the question “How much do you think people risk harming themselves if they gamble” with possible answers “No risk”, “Slight risk”, “Great risk” and “Don't know”.

Impulsiveness was measured through five items retrieved from the Eyseneck Impulsiveness scale (Eysenck and Eysenck, 1978), later on used by Vitaro et al. (1999): “I often say or do things without thinking”, “I often get in troubles because I do things without thinking it through”, “I am impulsive person”, “I weight up all the choices before I decide on something”, and “I often say something off the top of my head”. The reliability of the scale was good (Cronbach's alpha α = 0.77).

Finally, sensation-seeking was evaluated with the Italian version of Brief Sensation Seeking Scale (BSSS), including questions on experience seeking (e.g. “I would like to explore strange places”), boredom susceptibility (e.g. “I get restless when I spend too much time at home”), thrill and adventure seeking (e.g. “I like to do frightening things”), and disinhibition (e.g. “I like wild parties”) (Primi et al., 2011). The reliability of the scale was good (Cronbach's alpha α = 0.76).

Questions on parental monitoring, disappointment, support, beliefs and attitudes toward gambling, impulsiveness and sensation-seeking allowed response alternatives on a 4-point Likert scale (very likely/likely/unlikely/very unlikely and strongly agree/agree/disagree/strongly disagree). The answers on questions on parental monitoring, beliefs, attitudes, impulsiveness and sensation seeking were scored 1–4 and summed, means were calculated, and categories of high, middle, and low level of each indicator were created by using tertiles.

### 2.4 Statistical analysis

The main outcome under study was whether the adolescent had gambled in the last 12 months (yes/no).

Descriptive data were summarized as frequency and percentage for categorical variables and as mean and SD for continuous variables. The *p*-values for continuous variables were obtained through t-test, and through Pearson's Chi-square test for categorical variables.

The associations of sociodemographic characteristics, parental gambling, gambling with parents, parental permissiveness, parental monitoring, parental support, and normative perceptions of friend's gambling with the probability of adolescent's gambling in the last 12 months were estimated through multivariate regression models. Collinearity between variables were checked before building the final models. Non-collinear and statistically significant variables from the bivariate model were included in the final multivariate regression model simultaneously. Variables “Gambling with father”, “Gambling with mother”, “Gambling with siblings” and “Gambling with other relatives” were collinear. Due to the important effect of gambling with both parents on adolescents' gambling, two multivariable models were then built: the first including the variable “Gambling with mother”, whereas the second including the variable “Gambling with father”.

Multilevel mixed-effect modeling was used to control for the hierarchical nature of the data, with two grouping levels: center (NHS district) as I level, and class as II level. The LR test showed that adding the third level “school” did not make a statistically significant difference (*p*=0.7), so the two-level model was used. Some levels in the variable “Center” were merged because of similarities of the context and low sample size in NHS districts, so the final variable had four levels instead of nine, i.e., Rome, Torino3/Torino5/Torino, Vercelli/Cuneo1/Cuneo2, and Alessandria/Novara. Adjusted Odds Ratios (AORs) and 95% Confidence Interval (95%CI) were estimated as measures of association between the studied factors and the outcome. Categorical variables were re-coded in order to reduce the number of items included in the model, i.e., categories were merged. Due to the correlation between the two variables (r = 0.60) and for the purpose of building the multivariate model, “Parental permissiveness to smoke” and “Parental permissiveness to drink” were merged into a single variable “Parental permissiveness to use licit substances”. A tetrachoric correlation matrix was used to assess the correlations between the two permissiveness variables.

Mediation analysis was conducted to test the mediating effect of personal factors (beliefs, attitudes, risk perceptions, impulsiveness and sensation-seeking) on the relationship between parental behaviors (parental gambling, gambling with mother and gambling with father) and adolescents' last 12 months gambling behavior using the PROCESS macro for SPSS (Hayes, 2018). The multivariate mediation effect was tested adjusting for confounders (gender, age, parental income and center). The indirect effect and 95% Confidence Interval were obtained through bootstrapping (10,000).

Missing data were < 4% for all studied variables, except for “Gender” that had 5.6% of missing information. Applying listwise deletion to handle missing data, the final model was run on 88% of the initial sample.

Statistical analysis was carried out using STATA software release 18.0 and SPSS software release 28 (StataCorp, 2023; IBM Corp, 2021).

## 3 Results

The prevalence of pupils gambling in the 12 months preceding the survey was 55.7% overall (56.1% males, 54.0% females).

The sample originated from 9 different centers (NHS districts) in Northern (64.9%) and Central Italy (35.2%) (Table 1). The mean age of the students participating in the survey was 13.1 (± 0.8) years. About half of the sample were boys (51.9%). No statistically significant differences emerged with respect to gender, age, languages spoken in family, father's employment status, family composition and disappointing parents between those who have gambled (G) and those who have not gambled (NG) at least once in the previous year.

**Table 1:** Socio-demographic and family factors by gambling behavior in the last 12 months.

**Characteristics**	**Overall (*****N*** = **1,848)**		**No gambling (*****N*** = **819)**		**Gambling (*****N*** = **1,029)**		***p*-value**
	***N***	**%**	***N***	**%**	***N***	**%**	
**Center (NHS district)**							
Roma	650	35.2	238	29.1	412	40.0	< 0.001
Alessandria	397	21.5	177	21.6	220	21.4	
Torino 3	387	20.9	184	22.5	203	19.7	
Torino 5	52	2.8	23	2.8	29	2.8	
Vercelli	118	6.4	77	9.4	41	4.0	
Cuneo 1	98	5.3	52	6.4	46	4.5	
Cuneo 2	22	1.2	8	1.0	14	1.4	
City of Torino	84	4.6	36	4.4	48	4.7	
Novara	40	2.2	24	2.9	16	1.6	
**Gender**							
Female	840	48.1	386	49.3	454	47.2	0.382
Male	905	51.9	397	50.7	508	52.8	
**Age (years)**							
12	517	28.0	220	26.9	297	28.9	0.353
13	830	44.9	364	44.4	466	45.3	
14	501	27.1	235	28.7	266	25.9	
**Age (years)**							
Mean±SD	13.1	0.8	13.1	0.8	13.0	0.8	0.989
**Spoken languages in family**							
Only Italian	1,366	74.2	599	73.4	767	74.9	0.466
At least one other	474	25.8	217	26.6	257	25.1	
**Spoken languages in family**							
Italian/English/German/French	1,403	75.9	615	75.1	788	76.6	0.227
Spanish/Portoghese	72	3.9	33	4.0	39	3.8	
Arabian	78	4.2	45	5.5	33	3.2	
Slavic/Russian/Albanian	78	4.2	36	4.4	42	4.1	
Chinese/Indian/Philippines	40	2.2	18	2.2	22	2.1	
Other	177	9.6	72	8.8	105	10.2	
**Family holidays in the last year**							
More than twice	796	43.4	317	39.0	479	46.9	0.003
Twice	501	27.3	232	28.6	269	26.3	
Once	430	23.5	204	25.1	226	22.1	
Never	107	5.8	59	7.3	48	4.7	
**Father's employment status**							
Work	1,724	97.0	755	96.6	969	97.4	0.221
Doesn't work	41	2.3	23	2.9	18	1.8	
Retired	12	0.7	4	0.5	8	0.8	
**Mother's employment status**							
Work	1,430	79.9	610	77.2	820	82.0	0.012
Doesn't work	360	20.1	180	22.8	180	18.0	
**Parental income**							
Two salaries	1,360	74.4	576	71.3	784	76.9	0.005
One salary	446	24.4	217	26.9	229	22.5	
No salary	22	1.2	15	1.9	7	0.7	
**Money from parents**							
Almost never/Rarely	1,098	59.8	518	63.6	580	56.7	0.003
Sometimes/Often	738	40.2	296	36.4	442	43.3	
**Family composition**							
Both parents	1,438	78.0	635	77.7	803	78.2	0.762
One parent	193	10.5	90	11.0	103	10.0	
Others	213	11.5	92	11.3	121	11.8	
**Parents gambling**							
No	1,226	67.0	633	77.9	593	58.3	< 0.001
Yes	418	22.8	118	14.5	300	29.5	
Don't know	187	10.2	62	7.6	125	12.3	
**Gambling with father**							
Never in general/with my father	1,245	67.8	699	86.0	546	53.4	< 0.001
Sometimes	501	27.3	108	13.3	393	38.4	
Often	90	4.9	6	0.7	84	8.2	
**Gambling with mother**							
Never in general/with my mother	1,408	76.7	739	90.7	669	65.6	< 0.001
Sometimes	372	20.3	72	8.8	300	29.4	
Often	55	3.0	4	0.5	51	5.0	
**Gambling with siblings**							
Never in general/with my siblings	1,535	84.3	776	95.6	759	75.3	< 0.001
Sometimes	225	12.4	31	3.8	194	19.2	
Often	60	3.3	5	0.6	55	5.5	
**Gambling with relatives**							
Never in general/with my relatives	1,288	70.2	691	85.0	597	58.4	< 0.001
Sometimes	475	25.9	111	13.7	364	35.6	
Often	73	4.0	11	1.3	62	6.1	
**Parental permissiveness to drink alcohol**							
Would not allow at all	1,374	75.2	646	79.7	728	71.7	< 0.001
Would not allow at home/Would allow	453	24.8	165	20.3	288	28.3	
**Parental permissiveness to smoke**							
Would not allow at all	1,619	88.5	742	91.4	877	86.2	0.001
Would not allow at home/Would allow	211	11.5	70	8.6	141	13.8	
**Parental permissiveness to gamble**							
Would not allow	1,651	90.8	792	97.7	859	85.3	< 0.001
Would allow	167	9.2	19	2.3	148	14.7	
**Parental monitoring**							
High	899	49.3	435	53.8	464	45.7	< 0.001
Middle	571	31.3	246	30.4	325	32.0	
Low	354	19.4	128	15.8	226	22.3	
**For me is important to not disappoint my parents**							
Yes	1,700	92.9	758	93.6	942	92.4	0.346
No	129	7.1	52	6.4	77	7.6	
**Parental support**							
Yes	1,575	86.3	718	88.4	857	84.5	0.016
No	251	13.7	94	11.6	157	15.5	
**Perception of friends gambling**							
No	1,291	70.6	668	82.4	623	61.3	< 0.001
Yes	537	29.4	143	17.6	394	38.7	

SD, Standard deviation.

As regards socioeconomic status indicators, the number of holidays undertaken by the family in the last year appear to be significantly associated with gambling behavior among children (*p* = 0.003), as well as a higher parental income as measured through the number of salaries in the family (*p* = 0.005). No differences emerged for father's employment status, but a significantly greater proportion of adolescents in group G compared to group NG had a working mother (82.0% vs. 77.2%, *p* = 0.012). About 40% of adolescents received money from their parents, 43.3% of those who gambled in the previous year compared to 36.4% of those who have not gambled (*p*=0.003).

About 22% of the students reported that their parents gambled, 29.5% among G vs. 14.5% among NG, and the proportion was higher also among those who were unsure whether their parents gambled or not (*p* < 0.001). Adolescents who gambled reported that they gamble with parents, siblings or relatives to a greater extent compared to NG (*p* < 0.001). Adolescents who gambled more frequently perceived parental permissive attitudes toward alcohol (28.3% vs. 20.3%, *p* < 0.001), smoking (13.8% vs. 8.6%, *p* = 0.001) and gambling (14.7% vs. 2.3%, *p* < 0.001). Low parental monitoring and lack of parental support were declared by a significantly higher proportion of G compared NG students (22.3% vs. 15.8%, *p* < 0.001 and 15.5% vs. 11.6%, *p* = 0.016, respectively). G students had a higher perception of the prevalence of friends' gambling than NG students (38.7% vs. 17.6%, *p* < 0.001).

As regards adolescent personal factors, high performance beliefs toward gambling (37.0% vs. 16.8%, *p* < 0.001), high positive attitudes (40.0% vs. 13.9%, *p* < 0.001), low negative attitudes (37.1% vs. 24.7%, *p* < 0.001), low risk perceptions (32.8% vs. 20.0%, *p* < 0.001), high impulsiveness (35.7% vs. 27.9%, *p* < 0.001) and high sensation-seeking (37.4% vs. 24.7%, *p* < 0.001) were more prevalent among pupils in group G compared to NG (Table 2).

**Table 2:** Personal factors of the study sample by gambling behavior in the last 12 months.

**Characteristics**	**Overall (*****N*** = **1,848)**		**No gambling (*****N*** = **819)**		**Gambling (*****N*** = **1,029)**		***p*-value**
	***N***	**%**	***N***	**%**	***N***	**%**	
**Performance beliefs toward gambling**							
Low	724	39.9	425	53.1	299	29.5	< 0.001
Middle	581	32.0	241	30.1	340	33.5	
High	510	28.1	134	16.8	376	37.0	
**Positive attitudes on gambling**							
Low	931	51.0	531	65.8	400	39.3	< 0.001
Middle	375	20.6	164	20.3	211	20.7	
High	519	28.4	112	13.9	407	40.0	
**Negative attitudes on gambling**							
High	837	45.8	414	51.2	423	41.5	< 0.001
Middle	414	22.6	195	24.1	219	21.5	
Low	578	31.6	200	24.7	378	37.1	
**Risk perceptions toward gambling**							
High	529	28.6	284	34.7	245	23.8	< 0.001
Slight	488	26.4	200	24.4	288	28.0	
Low	501	27.1	164	20.0	337	32.8	
Don't know/missing	330	17.9	171	20.9	159	15.5	
**Impulsiveness**							
Low	759	41.6	388	48.0	371	36.5	< 0.001
Middle	478	26.2	195	24.1	283	27.8	
High	588	32.2	225	27.9	363	35.7	
**Sensation seeking**							
Low	745	41.1	399	49.6	346	34.3	< 0.001
Middle	493	27.2	207	25.7	286	28.3	
High	576	31.7	199	24.7	377	37.4	

In bivariate regression models, parental gambling, gambling with parents and other family members, parental permissiveness to drink alcohol, smoke and gamble, middle/low parental monitoring, low parental support, having received money from parents, and perception of friend's gambling were significantly associated with adolescent gambling. Low parental income, non-working mother and no/rare family holidays were associated with lower odds of adolescents' gambling (data not shown).

### 3.1 Multivariate regression model with “gambling with mother”

In the multivariate regression model including “gambling with mother” variable, several indicators (gender, age, received money from parents, parental monitoring and parental support) lost significance (Table 3).

**Table 3:** Correlates of adolescent's gambling in the last 12 months: multilevel multivariate regression models.

**Characteristics**	**AOR (95%CI)^a^ *N* = 1,620**	***p*-value**	**AOR (95%CI)^b^ *N* = 1,622**	***p*-value**
**Gender**				
Female	1		1	
Male	1.09 (0.87–1.38)	0.455	0.98 (0.78–1.24)	0.875
Age (cont)	0.93 (0.76–1.15)	0.501	0.94 (0.77–1.16)	0.587
**Parental income**				
Two salaries	1		1	
One salary/No salary	0.77 (0.59–1.01)	0.060	0.80 (0.61–1.03)	0.088
**Money from parents**				
Almost never/rarely	1		1	
Sometimes/Often	1.21 (0.95–1.54)	0.115	1.23 (0.97–1.56)	0.091
**Parents gambling**				
No	1		1	
Yes	1.62 (1.21–2.18)	**0.001**	1.33 (0.98–1.80)	0.069
Don't know	1.74 (1.16–2.63)	**0.008**	1.62 (1.08–2.43)	**0.021**
**Gambling with mother**				
Never in general/with mother	1			
Sometimes/often	4.52 (3.27–6.26)	**< 0.001**	–	
**Gambling with father**				
Never in general/with father			1	
Sometimes/often	–		3.90 (2.96–5.16)	**< 0.001**
**Parental permissiveness to use licit substances** ^c^				
Would not allow at all	1		1	
Would not allow at home/Would allow	1.40 (1.07–1.84)	**0.013**	1.33 (1.02–1.74)	**0.034**
**Parental permissiveness to gamble**				
Would not allow	1		1	
Would allow	3.67 (2.04–6.59)	**< 0.001**	3.37 (1.88–6.05)	**< 0.001**
**Parental monitoring**				
High	1		1	
Middle	1.15 (0.88–1.49)	0.299	1.18 (0.91–1.53)	0.223
Low	1.30 (0.94–1.81)	0.115	1.34 (0.97–1.86)	0.076
**Parental support**				
High	1		1	
Low	1.26 (0.89–1.79)	0.188	1.27 (0.90–1.80)	0.174
**Perception of friends gambling**				
No	1		1	
Yes	2.42 (1.85–3.17)	**< 0.001**	2.31 (1.77–3.02)	**< 0.001**

Multilevel mixed-effect models controlled for two levels: center and class.

Adjusted Odds Ratios (AOR); 95% Confidence Interval (95%CI).

^a^Multivariate regression model including the variable “Gambling with mother”.

^b^Multivariate regression model including the variable “Gambling with father”.

^c^Merged parental permissiveness to smoke and parental permissiveness to drink alcohol.

Statistically significant results are marked in bold (*p* < 0.05).

Gambling with mother (OR 4.52, 95%CI 3.27–6.26) and parental permissiveness to gamble (OR 3.67, 95%CI 2.04–6.59) were the strongest correlates of adolescents' gambling. Both parental gambling (OR 1.62, 95%CI 1.21–2.18), and not knowing if parents gambled (OR 1.74, 95%CI 1.16–2.63) were associated with gambling. The odds of gambling were higher among adolescents who perceived parental permissive attitudes toward the use of licit substances (OR 1.40, 95%CI 1.07–1.84). Adolescents who perceived their friends gambled had about twice greater odds of gambling themselves (OR 2.42, 95%CI 1.85–3.17). Low parental income was marginally associated with adolescents' gambling (OR 0.77, 95%CI 0.59–1.01).

### 3.2 Multivariate regression model with “gambling with father”

In the multivariate regression model including “gambling with father” variable, again, the indicators of gender, age, parental monitoring and parental support lost significance (Table 3).

Similarly, gambling with father (OR 3.90, 95%CI 2.96–5.16) and parental permissiveness toward gambling (OR 3.37, 95%CI 1.88–6.05) were the strongest correlates of adolescent gambling. However, as regards parental gambling, only not knowing if parents gambled was a significant correlate of adolescent gambling (OR 1.62, 95%CI 1.08–2.43). The odds of gambling were associated also with adolescents perceived parental permissive attitudes toward use of licit substances (OR 1.33, 95%CI 1.02–1.74). Perception of friends gambling was associated with adolescents' engagement in gambling (OR 2.31, 95%CI 1.77–3.02). Low parental income (OR 0.80, 95%CI 0.61–1.03), parental gambling (OR 1.33, 95%CI 0.98–1.80) and receiving money from parents (OR 1.23, 95%CI 0.97–1.56) were only marginally associated with adolescents' gambling.

### 3.3 Mediation model

**Figure 1:**
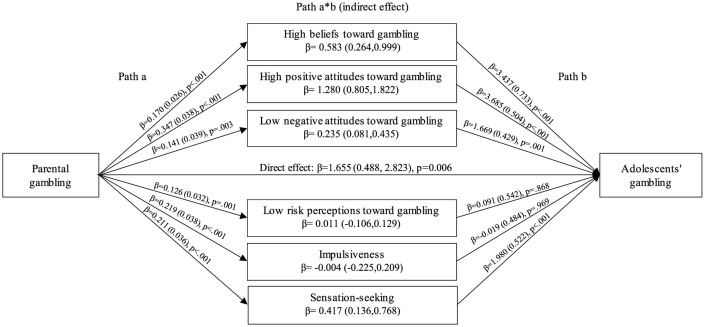
Mediators of parental gambling on last 12 months adolescent's gambling. Multivariate mediation model adjusted for age, gender, parental income and center. Path a: effect of parental gambling on targeted mediators. Path b: effect of targeted mediators on adolescents' gambling. Path a*b: indirect or mediation effect of targeted mediators.

Parental gambling was significantly associated with high performance beliefs toward gambling (*p* < 0.001), high positive attitudes toward gambling (*p* < 0.001), low negative attitudes toward gambling (*p* = 0.003), low risk perceptions toward gambling (*p* = 0.001), impulsiveness (*p* < 0.001) and sensation-seeking (*p* < 0.001) (Path a, Figure 1). High performance beliefs toward gambling, high positive attitudes toward gambling, low negative attitudes toward gambling and sensation-seeking suggested a potential partially mediated pathway between parental gambling and adolescents' gambling, as the direct effect remained significant (β = 1.655, *p* = 0.006). The same factors were associated with adolescents' gambling in a manner consistent with mediation in the models using gambling with mother and gambling with father as exposure (data not shown).

## 4 Discussion

The present study investigated parental factors related to gambling behaviors in early adolescents, as well as the mediating effect of adolescent factors on the relationship between parental gambling behaviors and adolescents' gambling. Parental gambling, gambling with parents, parental permissiveness to gamble and use licit substances, and perceived friends' gambling were significantly associated with higher odds of adolescent gambling. Adolescent performance beliefs, attitudes toward gambling, and sensation-seeking mediated the effect of parental gambling and gambling with parents on the probability of adolescent gambling.

The high prevalence of gambling behavior in our sample corroborates the early onset of gambling behavior (Gambling Commission, 2023; Derevensky et al., 2019; Westphal et al., 2000), highlighting the need of prevention interventions dedicated to early adolescents. The prevalence (55,7%) is higher than what previously observed in Italy both among 16-years old students participating in the ESPAD survey (32%) and among 15-years old students participating in the HBSC survey (37.5% among males and 14% among females) (ESPAD Group, 2020; HBSC, 2022). Socio-economic status, proportion of males and females, and of students with migratory background are comparable between our sample, ESPAD and HBSC participants, so the main difference appears to be the younger age of students of our sample, making these results particularly concerning. However, the higher prevalence of gambling behavior we observed could also be due to differences in recruitment procedures, sampling and timing. Our sample include only students of two Italian Regions, and of schools that voluntarily participated in the study, and whose parents provided consent for participation. The timing of data collection could also have impacted, since the ESPAD data were collected pre-COVID (2019), whereas our study took place post-COVID (2022/2023). Pandemic-related factors may have influenced adolescents' susceptibility to risk behaviors.

The early onset and the high percentage of underage individuals who engaged in gambling can be due to today widespread gambling opportunities, even in socially acceptable forms (Delfabbro and Thrupp, 2003). For example, in Italy, national lottery results are broadcasted on public television in a festive tone, potentially contributing to a perception of gambling as a normalized activity. Whereas these factors alone do not account for the high prevalence observed, they may contribute to a broader cultural and environmental context that deserves further investigation, especially regarding early prevention (Lupu and Todirita, 2013).

Although only marginally significant in multivariate model, gambling behavior appears to be associated with higher family socioeconomic status, consistently with some previous studies (Moñino-García et al., 2022; Buja et al., 2019; Welte et al., 2008). This association may be explained with a tendency of adolescents to spend money on gambling if available (Forrest and McHale, 2012) and can be favored by owning personal devices (smartphones, tablets, PCs) facilitating online gambling. Moreover, considering gambling as a type of financial behavior, it is conceivable that parents who emphasize savings, proper money management, and provide allowances for their children may encourage more responsible money usage and a more realistic approach to earnings, thereby mitigating gambling risks (Delfabbro and Thrupp, 2003). On the other side, the inverse association between low SES and gambling may also reflect specific contextual factors, such as limited gambling access and fewer technological resources in low-SES households. The relationship between SES and gambling is complex: lower SES can increase risk in some domain, whereas higher SES may relate to gambling as a leisure activity. Jessor's Problem Behavior Theory (PBT) posit that gambling results from the interaction between individual characteristics, behavioral tendencies, and environmental influences (Palomäki et al., 2025). Future research is needed to further explore the contextual mechanisms underlying the relationship between socioeconomic status and adolescent gambling.

Gambling with parents (both mother and father), and parental gambling were associated with adolescent gambling. The significant effect of parental gambling on adolescents' behavior is extensively recognized (Delfabbro et al., 2005; Donati et al., 2023; Gupta and Derevensky, 1997; Hardoon et al., 2004; Langhinrichsen-Rohling et al., 2004; McComb and Sabiston, 2010; Vachon et al., 2004). Parental behavior serves as a powerful role model for children shaping their beliefs, attitudes, and behaviors through observational learning and imitation (Bandura, 1977, 1986; Gupta and Derevensky, 1998). The social learning theory highlights the interaction between personal and environmental factors, explaining how parental influence shapes both adolescents' actions and their risk-taking tendencies, including gambling (Canale et al., 2016; Savard et al., 2015). Moreover, parental involvement in gambling behavior may convey a harmless image of gambling, favoring the perception of gambling as acceptable activity, and fostering positive attitudes toward gambling which in turn increase the likelihood of child's first engagement in gambling (Griffiths and Wood, 2000; Hanss et al., 2014; Pallesen et al., 2016). Notably, in our study adolescents who were uncertain about their parents' gambling had higher odds of engaging in gambling themselves. This may reflect limited family communication or parental monitoring, which may increase vulnerability to risky behaviors. Coherently, in our study parental permissiveness toward gambling was associated with greater adolescent engagement in gambling activities, as already observed in previous studies (Delfabbro and Thrupp, 2003; Hardoon et al., 2004; Wickwire et al., 2010; Leeman et al., 2014). Moreover, not only parental permissiveness to gamble had a significant effect on adolescent gambling behavior, but also permissiveness to use licit substances (alcohol and tobacco). This finding suggests that parental influence is not limited to a single risk behavior, but it can generalize to various risk behaviors. Several other studies found that parental approval of one substance can influence the use of different substances (Li et al., 2002; Koning et al., 2020; Mehanović et al., 2022).

The normative role of perceived friends' gambling was associated with engaging in gambling, aligning with the substantial existing literature (Renna et al., 2025; Casey et al., 2011; Vegni et al., 2019; Pallesen et al., 2016; Langhinrichsen-Rohling et al., 2004; Delfabbro and Thrupp, 2003; Hardoon et al., 2004; Mazar et al., 2018). Adolescents' subjective perception of norms may not accurately reflect peers' actual gambling behavior, since can be subject to cognitive biases. However, it is recognized that among adolescents, it may even be more impactful on the adoption of risk behaviors than the actual norms (Elek et al., 2006; Perkins et al., 2019).

Finally, we investigated paths that may explain the association of parental factors with adolescents' gambling. Through mediation models, we found that both parental gambling and gambling with parents were associated with their child's performance beliefs and positive attitudes toward gambling, which were in turn associated with adolescent gambling. Therefore, adolescents' own beliefs and attitudes, recognized risk factors for gambling behavior (Derevensky and Gilbeau, 2015; Griffiths and Wood, 2000; Hanss et al., 2014; Hurt et al., 2008; Pallesen et al., 2016), were potentially modeled by their parental behavior. High sensation-seeking, a well-known risk factor for adolescent gambling (Hurt et al., 2008; Dowling et al., 2017; Derevensky and Gilbeau, 2015) was another potential mediator in the above-mentioned relationship. This again underscores the role of parental behaviors in shaping adolescents' personal characteristics, attitudes, and skills, influencing their risk behaviors (Canale et al., 2016). Understanding these dynamics can help prevention practitioners to develop appropriate interventions to promote healthier decision-making in children. On the contrary, in our study impulsiveness and risk perceptions were not statistically significant as potential mediators of the relationship between parental and adolescent gambling. So, we can confirm certain cognitive and personality traits (sensation-seeking and gambling-related attitudes) as potentially mediating factors, whilst the role of others appears to be less clear.

The results of our study suggest that, whereas interventions at the broader societal level are essential, certain family-related aspects should be enhanced, particularly by raising parents' awareness. Undoubtedly, a parental stance explicitly opposed to any form of gambling can serve as a protective factor. A more attentive monitoring of the adolescents, without compromising their autonomy, has also demonstrated effectiveness (Floros et al., 2013). Additionally, instilling the values of responsible money management is highly recommended (Delfabbro and Thrupp, 2003).

Prevention programs should actively engage parents to enhance their awareness and responsibility. Feasible strategies may include school-based workshops aimed at educating parents about gambling risks and their role-model influence, media campaigns addressed to parents, as well as general media campaigns aimed to raise broader public awareness. These interventions should be focused to enhance parental monitoring and communication, and to strengthen protective factors against youth gambling.

In research, the development and the use of standardized validated tools should be promoted to objectively measure parental factors and parenting styles. Furthermore, more research should be conducted to compare adolescents' subjective perceptions of parental behavior with parents' self-reported responses.

Finally, our study measured gambling activity rather than gambling-related harm. Early gambling activity is a known risk factor for later harm and may signal risk (Volberg et al., 2010), however, it does not necessarily imply a future development of problematic patterns. More studies are needed to explore the progression from gambling activity to gambling harm to better tailor prevention efforts.

This study has several strengths. The survey used a standardized questionnaire containing previously validated questions derived from recognized international sources, minimizing possible misclassifications related to data collection and measures. Multilevel mixed-effect regression models were performed to evaluate the association between correlates and gambling, according to higher order clustering (center and class). The information collected through the questionnaires allowed the analysis of a large set of correlates.

However, the study has also limitations. The cross-sectional nature of the study prevents inferring causality. Indeed, some variables included in the analyses are likely to precede the outcome, e.g., sociodemographic characteristics, family composition and parental gambling, but for most other variables, including parental factors, bidirectional or reverse causality cannot be excluded. Missing values reduced the sample in the adjusted regression models; however, the models were run on 88% of the sample, a value that should maintain low the risk of attrition bias. The sample was not equality distributed between the nine NHS districts; therefore, it was needed to merge some to perform multilevel analysis. All the information was self-reported by the students; therefore, the student's subjective perceptions of their friends' gambling, parent's gambling and permissiveness may not accurately reflect the reality. Nevertheless, students' perceptions may have a strong impact on their behavior. The proxies of SES used in this study (family holidays and parental occupational status) may not fully distinguish the financial and social capital of the families, so limiting the reliability of the association between SES and adolescent gambling we observed. Finally, despite recruiting participants from schools located in different geographic areas, the possibility of selection bias cannot be ruled out. The sample includes indeed only students who participated in the trial and whose parents provided informed consent. It is possible that the participating schools were those most engaged in prevention, or those where the issue was more strongly perceived. Parents from different socio-economic backgrounds or with varying attitudes toward gambling may have declined to give consent for various reasons. All these reasons can limit the generalizability of the findings.

In conclusion, parental gambling and gambling with parents were significantly associated with adolescents' beliefs, attitudes, and sensation-seeking, and these in turn were related to the probability of gambling behavior. Parental permissiveness toward gambling and licit substance use was associated with adolescent engagement in gambling activities, corroborating the need for intervention efforts on parental awareness. This study provides insights into the complex dynamics influencing adolescent gambling behavior and emphasize the importance of targeted interventions and parental guidance in promoting healthier decision-making and mitigating adolescent gambling problems.

## Data Availability

The raw data supporting the conclusions of this article will be made available by the authors, without undue reservation.
